# Disseminated adrenal histoplasmosis in an immunocompetent adult from Nepal: a case report

**DOI:** 10.1097/MS9.0000000000001240

**Published:** 2023-09-05

**Authors:** Aadhar Oli, Shila Poudel, Abirodh Ranabhat

**Affiliations:** a Kathmandu Medical College and Teaching Hospital, Kathmandu; b Manipal College of Medical Sciences, Pokhara, Nepal

**Keywords:** adrenal histoplasmosis, case report, disseminated histoplasmosis, histoplasmosis

## Abstract

**Introduction:**

Histoplasmosis is caused by the environmental fungus *Histoplasma capsulatum*. In immunocompromised patients, histoplasmosis can present as a disseminated infection that can involve the liver, lymph nodes, lungs, and adrenal glands. Disseminated histoplasmosis affecting the adrenal glands in an immunocompetent individual is a rare infection.

**Case presentation:**

A 53-year-old male without HIV complained of weight loss of 15 kg in 6 months. Computed tomography (CT) scan of the abdomen was done, which showed bilateral adrenal gland hyperplasia with hepatosplenomegaly. Endoscopic ultrasound fine needle aspiration of the adrenal gland revealed numerous budding yeast forms of Histoplasma intracellularly within the macrophages. A diagnosis of disseminated adrenal histoplasmosis was made. Liposomal amphotericin B and itraconazole therapy was started.

**Discussion:**

Disseminated histoplasmosis is commonly present in immunocompromised individuals. Immunocompetent individuals may also present with the disseminated form of the disease, which typically involves the adrenal glands. CT scan of the abdomen shows bulky adrenal glands with normal configuration, peripheral enhancement, and central hypodensities due to necrosis and/or hemorrhage. This might be a common presentation in other disseminated infections. Therefore, a definitive visualization of *H. capsulatum* in tissue specimens is the best method to confirm the diagnosis in a patient.

**Conclusion:**

Physicians must be suspicious of disseminated adrenal histoplasmosis in patients presenting with enlarged adrenal glands, even in immunocompetent individuals who are from endemic regions. Histopathological or cytological evaluation is the best method to establish a diagnosis.

## Introduction

Highlights

- Disseminated adrenal histoplasmosis in an immunocompetent patient is rare.
- Bilaterally enlarged adrenal glands are seen on imaging.
- Histopathological/cytological evaluation is the best method to establish a diagnosis.

Histoplasmosis is caused by a dimorphic fungus *Histoplasma capsulatum*. Histoplasmosis is endemic in North America and Latin America. It is more prevalent in the moist Ohio and Mississippi River valleys, where the soil is enriched with bat guano or bird droppings^1^. Humans are infected through various mechanisms, but most commonly, spores are inhaled into the alveoli. These spores then germinate into yeast and incite an immunological response. The yeasts are taken up by the alveolar macrophages, where Histoplasma replicates. Macrophages assist in spreading the organism throughout the reticuloendothelial system (liver, spleen, bone marrow, and lymph nodes). Most infections are asymptomatic and self-limiting. Symptomatic pulmonary infection can occur in immunocompetent individuals with substantial exposure to *H. capsulatum*. Acute pulmonary infection is more common in pediatric age groups. Disseminated histoplasmosis is more common in immunocompromised individuals^2–4^. Herein, we report a case of a 53-year-old immunocompetent male with delayed presentation of disseminated histoplasmosis in both adrenal glands, diagnosed through histopathological evaluation of the adrenal gland and treated with liposomal amphotericin B and itraconazole. This case report has been reported in line with the SCARE (Surgical CAse REport) Criteria^5^.

## Case report

**Figure 1:**
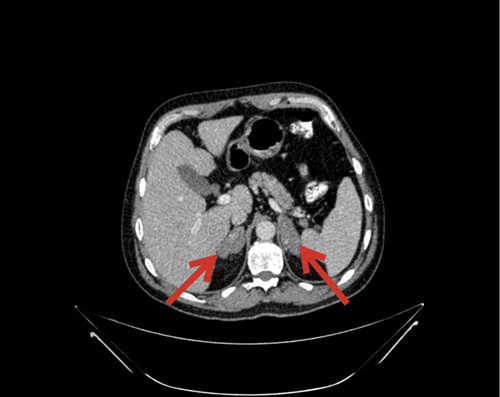
CT scan of the abdomen showing hepatosplenomegaly with bilateral adrenal hyperplasia.

**Figure 2:**
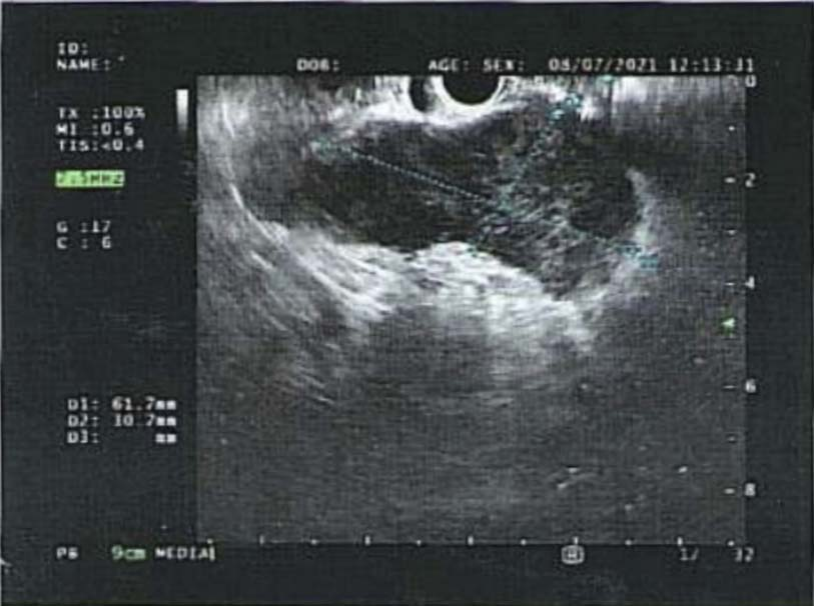
Endoscopic Ultrasound of the abdomen showing bulky adrenal gland.

**Figure 3:**
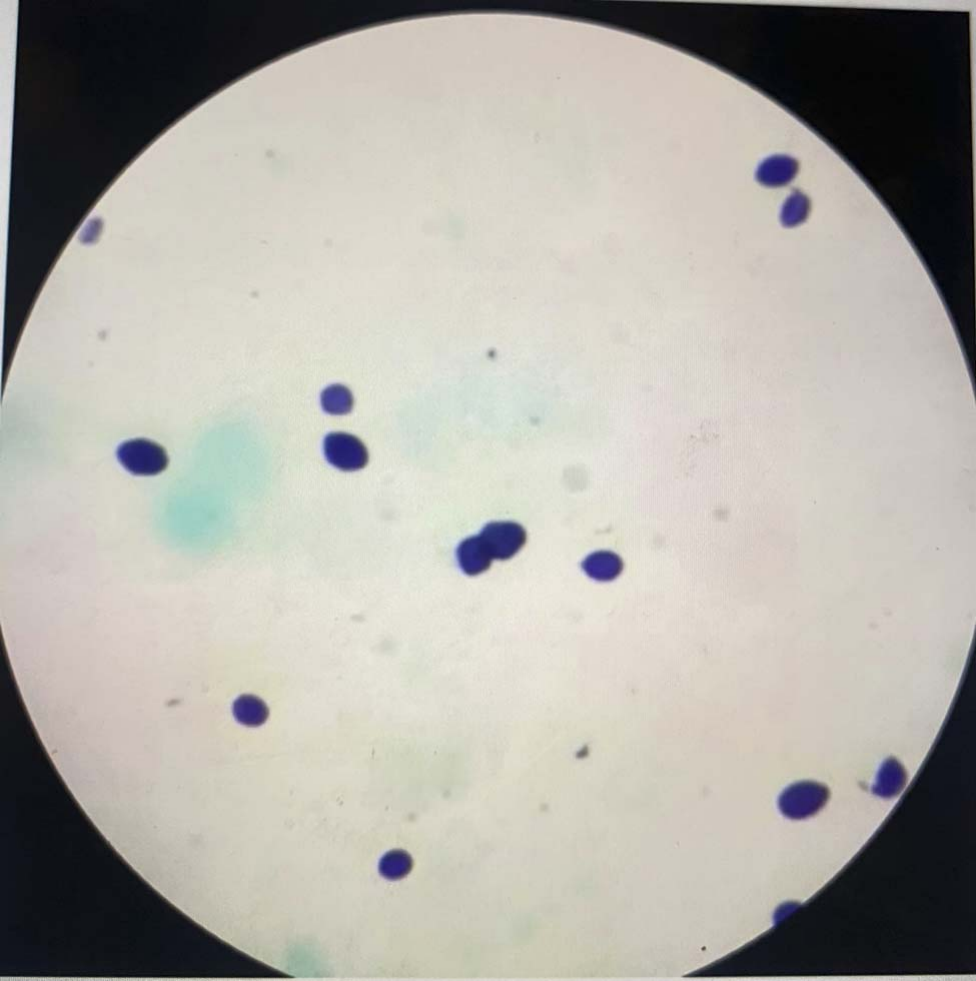
Fungal stain highlighting Histoplasma.

An HIV-negative male in his early 50s presented with a history of unintentional weight loss (15 kg in 6 months). His past medical history was only significant for hypertension, dyslipidemia, and gastroesophageal reflux disease. On further evaluation, he did not have any history of travel, exposure to bird or bat droppings, use of alcohol, or unsafe sex. His vitals, physical examination, and relevant labs [CBC (complete blood count) with differential, BMP (basic metabolic panel), AM cortisol (morning cortisol), and ACTH (adrenocorticotropic hormone) stimulation] were all within normal limits, which ruled out adrenal insufficiency secondary to destruction of the adrenal glands. Suspecting malignancy, a computed tomography (CT) scan of the abdomen was done, which revealed hepatosplenomegaly with bilateral adrenal hyperplasia. Both the adrenal glands were diffusely bulky and hypodense with heterogeneous post-contrast enhancement. The right adrenal gland measured 5.3×3.3 cm, and the left measured 6.3×5 cm. (Fig. 1). The liver was enlarged, measuring 16 cm, and the spleen was enlarged, measuring 14 cm. An endoscopic ultrasound of the abdomen was done, which also showed hypoechoic and heterogeneous bilateral bulky adrenal glands (Fig. 2). Transgastric fine needle aspiration (FNA) was done and the sample was sent for cytology, Gene Xpert, tuberculosis (TB) culture, and immunohistochemistry evaluation. Gene Xpert for TB was negative, and no acid-fast bacilli indicating TB was isolated after 42 days of incubation. On histopathological evaluation, numerous budding yeast forms of Histoplasma were seen intracellularly within the macrophages and in the background. The fungal stain highlighted numerous fungal organisms consistent with Histoplasma (Fig. 3). Upon the diagnosis of adrenal histoplasmosis, liposomal amphotericin B was started.

### Treatment

Liposomal amphotericin B 200 mg/day was started for 7 days, followed by itraconazole 200 mg twice a day for 1 year. On his subsequent visits, he was asymptomatic, gained weight, and tolerated his treatment well. Repeat CT scans showed a marked reduction in the sizes of both adrenal glands.

## Discussion

Histoplasmosis is usually an asymptomatic and self-limiting infection. Clinical manifestations of the disease depend on the host’s immune system and also the degree of exposure^6^. The disseminated form of the disease is more common in immunocompromised hosts (transplant recipients, HIV infection, immunosuppressive disorders)^7^. Though endemic in Latin America and North America, there are only a limited number of cases identified in Nepal^8^. A disseminated form of the disease in an immunocompetent individual, as in our case, is reported only occasionally^4,8,9^. In the disseminated form of the disease, the liver, spleen, lymph nodes, bone marrow, and adrenal glands are more commonly involved. This results in a wide variety of clinical presentations. Fever, weight loss, anorexia, nausea, vomiting, malaise, and fatigue are some of the more common presentations^3,6^. Physical examination may also reveal hepatosplenomegaly, lymphadenopathy, and skin nodules^6^. Our patient only presented with the clinical signs of weight loss. Adrenal involvement is a common presentation in immunocompetent patients^10,11^. Adrenal involvement may be unilateral or bilateral. The symptoms of adrenal insufficiency, like fever, malaise, orthostatic hypotension, nausea, vomiting, night sweats, hyperkalemia, hyponatremia, eosinophilia, and hyperpigmentation may be present in chronic infection due to atrophy and calcifications of the adrenal glands but are uncommon^6,10,12^. An abdominal CT scan typically shows an enlarged liver, spleen, and adrenal glands. The adrenal glands in CT scans are bulky with normal configuration, peripheral enhancement, and central hypodensities due to necrosis and/or hemorrhage^12,13^. Bilateral enlarged adrenal can be a common finding in adrenal hemorrhage, lymphoma, and metastatic or disseminated infections (histoplasmosis, TB, aspergillosis)^12^. Cytology or histopathological evaluation of the adrenals is therefore the best method to confirm the diagnosis. Examination of the tissue after a FNA or percutaneous biopsy will reveal typical features of *H. capsulatum*. Since *H. capsulatum* is an intracellular dimorphic fungus, narrow-based budding yeast can be seen in the macrophages and the cytoplasm^6,12^. Special stains like methenamine silver or periodic acid-Schiff stains can better visualize the yeast^6^. Lipid formulation of amphotericin B is the recommended initial management for moderate to severe disseminated histoplasmosis due to more rapid clearance of fungemia^14^. Therapy is then switched to itraconazole once the condition improves. Patients generally require treatment for at least a year to reduce the risk of relapse. Patients with immunocompromised state/immunosuppression require long-term/lifelong therapy to reduce the risk of relapse^15,16^.

## Conclusion

Disseminated histoplasmosis, though more common in immunocompromised individuals, can present in an immunocompetent individual which can involve the liver, spleen, lymph nodes, bone marrow, and adrenal glands. This case demonstrates that when faced with a patient with bilateral adrenal gland mass/hyperplasia, physicians should have a suspicion of adrenal histoplasmosis, especially in endemic areas. Histopathological or cytological evaluation can confirm the diagnosis so that timely and appropriate treatment can be provided to avoid complications.

## Ethical approval

Since this is a case report and not an original research article, IRC Norvic International Hospital, Kathmandu, Nepal, does not require ethical approval for case reports.

## Consent

Written informed consent was obtained from the patient for the publication of this case report and accompanying images. A copy of the written consent is available for review by the Editor-in-Chief of this journal on request.

## Sources of funding

This research did not receive any specific grant from funding agencies in the public, commercial, or not-for-profit sectors.

## Author contribution

All authors were involved in the writing of the paper, collecting data, revising it critically for important intellectual content, reviewing, and editing.

## Conflicts of interest disclosure

There are no conflicts of interest.

## Research registration unique identifying number (UIN)

This is not applicable since this is not an original article or a clinical trial and only a case report.

## Guarantor

Aadhar Oli, Kathmandu Medical College and Teaching Hospital, Kathmandu, Nepal; E-mail: adhar.olee@gmail.com.

## Provenance and peer review

Not commissioned, externally peer-reviewed.

## Data availability statement

Not applicable.

## Acknowledgements

Not applicable.
